# Majorana fermions in ferromagnetic chains on the surface of bulk spin-orbit coupled *s*-wave superconductors

**DOI:** 10.1038/srep08880

**Published:** 2015-03-06

**Authors:** Hoi-Yin Hui, P. M. R. Brydon, Jay D. Sau, S. Tewari, S. Das Sarma

**Affiliations:** 1 Department of Physics, Condensed Matter Theory Center and Joint Quantum Institute, University of Maryland, College Park, Maryland 20742, USA

## Abstract

Majorana fermion (MF) excitations in solid state system have non-Abelian statistics which is essential for topological quantum computation. Previous proposals to realize MF, however, generally requires fine-tuning of parameters. Here we explore a platform which avoids the fine-tuning problem, namely a ferromagnetic chain deposited on the surface of a spin-orbit coupled *s*-wave superconductor. We show that it generically supports zero-energy topological MF excitations near the two ends of the chain with minimal fine-tuning. Depending on the strength of the ferromagnetic moment in the chain, the number of MFs at each end, *n*, can be either one or two, and should be revealed by a robust zero-bias peak (ZBP) of height 2 *ne*^2^/*h* in scanning tunneling microscopy (STM) measurements which would show strong (weak) signals at the ends (middle) of the chain. The role of an approximate chiral symmetry which gives an integer topological invariant to the system is discussed.

Majorana fermions (MFs), which are also their anti-fermions by definition, were originally introduced almost 80 years ago in the context of understanding neutrinos. As real solutions of the Dirac equation they are therefore self-conjugate, thus representing both particles and anti-particles by the same real wavefunction. Whether neutrinos are MFs or not is still an open question being vigorously investigated in high-energy physics. A completely novel incarnation of MFs appeared more recently^1^,^2^,^3^ in condensed matter physics as zero-energy neutral bound states in the subgap excitation spectrum of topological superconductors (TS). The exact particle-hole symmetry characteristic of the excitation spectrum in superconductors usually sharply distinguishes states as either electron-like or hole-like in accordance with whether their energy is positive or negative, respectively. In *s*-wave superconductors the fermion-doubling theorem prevents the appearance of any zero-energy subgap excitations, and so MFs can only appear in effectively spinless *p*-wave superconductors, which are the canonical example of TS with a bulk gap and topologically protected gapless edge states. Such Majorana bound states (i.e. MFs in TS) in low-dimensional condensed matter systems obey anyonic non-Abelian braiding statistics and are in general anyons, not ordinary fermions, which makes them ideal for fault-tolerant quantum computation^1^,^2^,^3^. These MFs in TS systems typically arise at defect sites (e.g. vortex cores, interfaces and edges) as localized excitations, and are topologically protected against local perturbations by the bulk superconducting gap. Although spinless *p*-wave superconductors do not seem to exist in nature, there have been many recent proposals^4^,^5^,^6^,^7^,^8^,^9^,^10^,^11^,^12^ for artificially creating two- and one-dimensional systems which behave effectively as spinless *p*-wave superconductors which support MFs at their boundaries. There are even experimental claims of the possible observation^13^,^14^,^15^,^16^,^17^,^18^ of MFs in spin-orbit-coupled semiconductor-superconductor heterostructures following theoretical proposals^7^,^8^, but the situation is not definitively conclusive. Given the great fundamental and practical significance of MFs, it is desirable to have platforms where MFs could easily emerge for experimental observation and investigation.

In this paper we study theoretically a relatively simple scheme for realizing MFs in a condensed matter setting, involving ferromagnetic (presumably metallic, e.g. Fe or Co or Ni) chains placed on the surface of standard superconductors (e.g. Pb or Nb or Al) in an STM-type measurement set up^19^. The advantage of this scheme is that MFs in the proposed system generically occur in a wide range of parameter space, thus requiring little fine-tuning of system parameters (e.g. tuning the applied magnetic field appropriately as in the semiconductor heterostructure scheme^7^,^8^) although for some values of the parameters more than one MFs are spatially superimposed on each other. Consequently, a zero-bias tunneling peak (ZBP), which is a hallmark of the zero-energy MFs^20^,^21^, is robust and generic in the proposed system. Our work significantly extends the robustness of previous proposals to realize MFs in superconductor-ferromagnet heterostructures^9^,^10^, thus making such devices an attractive alternative to spin-orbit-coupled semiconductor-superconductor platforms.

In a conventional *Z*_2_ topological superconductor^22^,^23^,^24^,^25^, e.g., the spin-orbit-coupled semiconductor-superconductor heterostructure^7^,^8^, a pair of MFs spatially superimposed on each other mix and split to finite energies, thus essentially becoming low-energy fermionic subgap states^26^. In this class of TS systems, therefore, the number of MFs (*n*) at any point in space can be either zero or one. This topological restriction on *n* results in a greatly reduced parameter space in which to look for experimental signatures of MFs. In the semiconductor-superconductor nanowire heterostructure, for example, ZBPs are expected in the presence of an externally applied magnetic field – the so-called semiconductor Majorana wire – only when the number of semiconductor bands crossing the Fermi energy is odd^27^,^28^, a condition difficult to control experimentally. Similarly, proposals for realizing a Majorana fermion in ferromagnet-superconductor heterostructures^9^,^10^ have the stringent requirement that only one of the spin-split bands in the ferromagnet has a Fermi surface. In the experimental system we explore in this paper, strictly speaking, there is no topological restriction on the number of MFs that can be localized at a given point in space. As we show below, the absence of a restriction on *n*, resulting from a topological chiral symmetry^22^,^23^,^24^,^25^, results in a greatly enhanced parameter space in which MFs are realized. We emphasize, however, that only when *n* is odd does the Majorana multiplet follow non-Abelian braiding statistics although a robust ZBP in STM experiments should occur generically for any value of *n*. Of course, for the purpose of establishing topologically protected degenerate states that may be used to establish non-Abelian braiding^3^, it is necessary for the Majorana to be non-degenerate i.e. *n* = 1, and therefore the generic ZBP signature here cannot necessarily be identified with a non-Abelian Majorana “particle”. Our conceptual new finding that robust Majorana fermions may reside generically (i.e. with no fine-tuning) in superconductor-ferromagnet heterostructures, protected by a hitherto undiscovered chiral symmetry, is the important new result presented in this theoretical work.

## Results

### Ferromagnetic chain on a spin-orbit coupled superconductor

**Figure 1:**
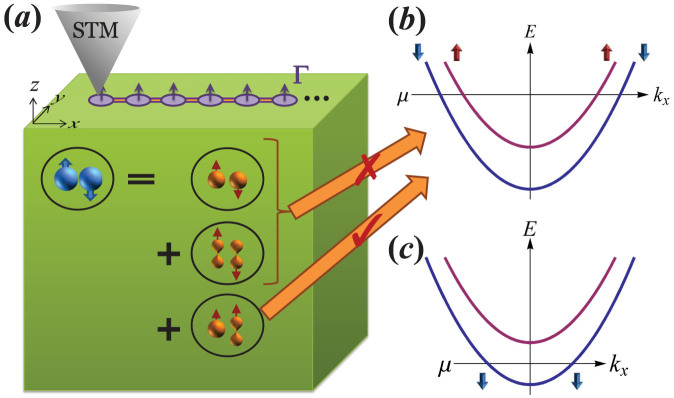
(a) Schematic diagram of our device. A ferromagnetic (FM) chain is placed on the surface of an *s*-wave superconductor, in which strong spin-orbit coupling and mixing of orbitals of opposite parity produce a pairing state with intra-orbital spin-singlet Cooper pairs and inter-orbital spin-triplet pairs. Tunneling of these pairs into the chain generates effective spin-singlet and spin-triplet pairing potentials, respectively, as shown in (b). For a FM chain with spin-splitting that exceeds the spin-singlet pairing potential, only the induced triplet pairing potential can gap the spectrum. In this case the system is in a topologically nontrivial state characterized by two unhybridized Majorana fermions at each end, which can be imaged by scanning tunneling microscopy (STM). When the FM chain is in the half-metal regime as shown in panel (c), however, only a non-Abelian single Majorana fermion is realized at each end. If the spin-splitting of the chain states is much smaller than their bandwidth, however, the situation (b) dominates the parameter space.

**Figure 2:**
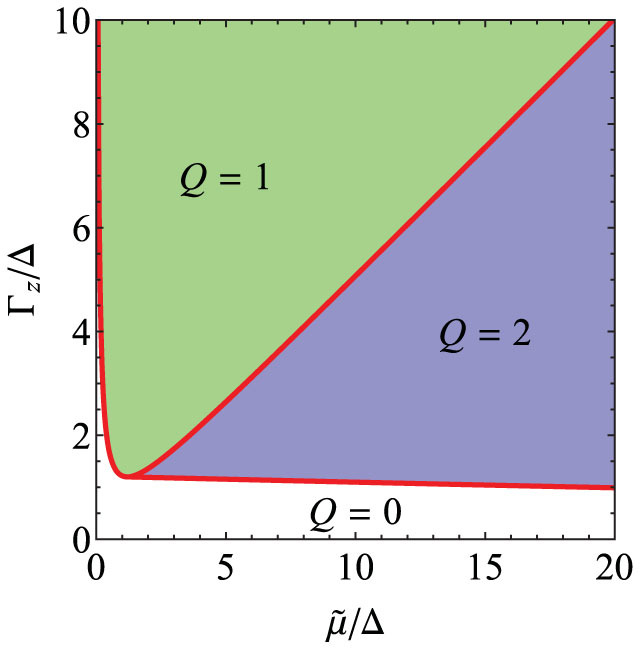
Topological phase diagram of the chain. The BDI topological index *Q* is defined in equation (8) calculated for 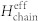 as a function of the Zeeman splitting Γ*_z_* and the chemical potential. The Green region (roughly 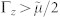) has *Q* = 1 while the blue region (roughly 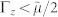) has *Q* = 2, indicating the existence of one and two Majorana fermions at each end of the chain, respectively.

**Figure 3:**
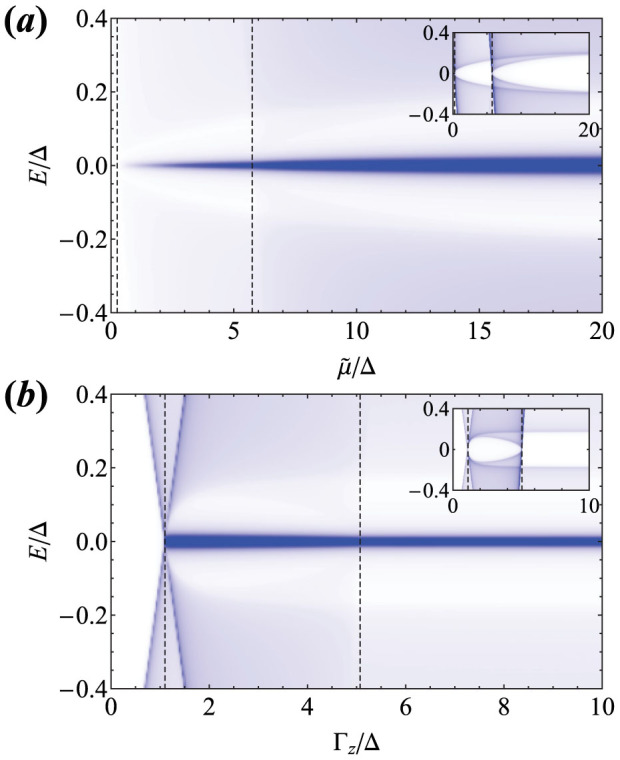
(a) The local density of states (LDOS) at the ends of the semi-infinite ferromagnetic (FM) chain as a function of the chemical potential 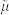 from the bottom of the bands, for Γ*_z_* = 3Δ. The LDOS has a strong zero-energy peak that for roughly 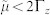 indicates a single Majorana fermion (MF) from the chain ends, while for 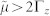 the zero-bias peak implies a pair of MFs localized at each ends protected by chiral symmetry. (b) the LDOS at the chain ends as a function of the Zeeman splitting for 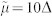. For roughly 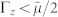 (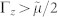) the zero-energy peak in LDOS signifies two (one) MFs at each end that can be accessed in scanning tunneling microscopy experiments. The insets shows the LDOS at the middle of the chain, which has a spectral gap in the topological regions. We indicate the transitions between the different topological sectors by the vertical dashed lines, and use arbitrary units for the LDOS in these plots.

A ferromagnetic (FM) chain (e.g. Fe), which is a single atom in width (although a few atoms should work too), is placed on the surface of a bulk *s*-wave superconductor, as shown schematically in Fig. 1. We emphasize that, in contrast to arrays of magnetic atoms on the surface of a superconductor, the FM chain is expected to have a bandwidth that is orders of magnitude larger than the superconducting pairing potential. We ignore the spin-orbit coupling within the FM chain (which plays no role in the scheme whether it exists or not, in sharp contrast to the semiconductor heterostructure-based Majorana schemes), but instead account for the existence of strong inversion-symmetric spin-orbit coupling in the bulk of the host superconductor. By integrating out the bulk superconductor we show that the effective Hamiltonian of the FM chain [equation (5) below] is in the chiral BDI class with an integer invariant, allowing an integer number *n* of MFs localized at the chain ends. If the FM chain has only one pair of spin-split sub-bands, *n* can be equal to zero, one, or two, but for *any* non-zero *n* (a condition that is realized in most of the parameter space (Fig. 2)) STM measurements at the chain ends should reveal a pronounced ZBP. The ZBP is in fact generic in our model, occurring in a wide region of the experimentally-accessible parameter space as shown in Fig. 2 and 3. No such peak is expected from the regions of the chain away from the ends where the MFs are localized. In practice the effective chiral symmetry in the FM chain should only be approximate, resulting in a finite energy width of the ZBPs for *n* > 1.

### Effective Hamiltonian of the chain

The superconductor used in our device must satisfy two key conditions: (i) there is strong spin-orbit coupling^29^, although inversion symmetry is not necessarily broken in the bulk, and (ii) orbitals of different parity both make a significant contribution to the states near the Fermi surface. The requirement that the orbitals have opposite parity can be relaxed, but this condition makes the following argument more transparent. The first condition implies that spin is not a good quantum number in the superconductor, but the presence of time-reversal (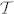) and inversion (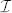) symmetry means that the doubly-degenerate eigenstates at each momentum **k** can be labeled by a pseudospin index *ς* = ±, such that 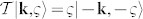 and 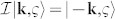. A conventional *s*-wave superconducting gap then corresponds to a pseudospin-singlet pairing state. To satisfy the second condition, we assume that the states near the Fermi surface are composed from two orbitals, say *s* and *p*, which are symmetric and antisymmetric under inversion, respectively. The general form of the pseudospin state is then
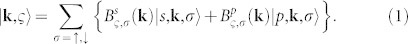
Due to the different parities of the two orbitals, the coefficients of the *s* and *p* states are even and odd in **k**, respectively. Expressed in the orbital basis using equation (1), one generally finds that the pseudospin-singlet pairing potential includes both intra-orbital spin-singlet and inter-orbital spin-triplet terms. The latter play a critical role in generating the topological state in the magnetic chain.

The tunneling between the magnetic chain and the superconductor is assumed to be local and independent of spin, and is therefore most transparently formulated in terms of tunneling between the chain atoms and the adjacent orbitals of the superconductor. We assume the form
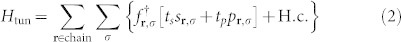
where *t_s_* and *t_p_* are the tunneling matrix elements for the two orbitals, assumed real, and *f***_r_**_,*σ*_, *s***_r_**_,*σ*_ and *p***_r_**_,*σ*_ are the annihilation operators for the site **r** in the chain and in the superconductor's *s* and *p* orbitals, respectively. The tunneling Hamiltonian implicitly accounts for the surface inversion-symmetry breaking: if the odd-parity orbital is odd with respect to mirror reflection in the surface plane, then tunneling from the chain sites into both the even- and odd-parity orbitals of the underlying superconductor can have a local component (see Fig. (1)). Since we are interested in the physics of the chain, our strategy is now to “trace out” the superconductor from the description of the problem. After standard manipulations as detailed in the Methods section, we obtain the self-energy correction for the chain
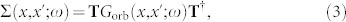
where the matrix **T** describes the tunneling between the chain and the superconductor, while *G*_orb_(*x*, *x*′; *ω*) is the Green's function of the superconductor expressed in the orbital-spin basis, and is related to the pseudospin Green's function by
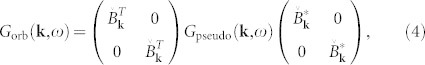
where 
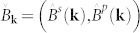
 is the matrix of coefficients in equation (1). The self-energy contains a complicated proximity-induced pairing term. Crucially, we find a *p*-wave spin-triplet pairing due to the tunneling of inter-orbital Cooper pairs from the superconductor. We generally expect that there will be triplet Cooper pairs with spin parallel to the magnetization, and so a gap appears in the spin-split states of the FM chain, see Fig. 1(b). By contrast, the spin-singlet pairing due to tunneling of intra-orbital Cooper pairs is unable to overcome the large exchange splitting. The proximity effect also renormalizes the bare dispersion and produces a spin-orbit coupling, but these effects are small and will be ignored.

The general expressions for the self-energy is unenlightening and presented in the Methods section. To make progress we write the general forms of the coefficient matrices 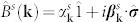 and 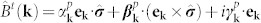, where **e_k_** is the unit vector in the direction of **k**, and the coefficients are all real and even functions of the momentum. The presence of mirror symmetries along three directions in the bulk, and two on its surface, further constrains the possible forms for *B^s^* and *B^p^*. To be concrete, we choose 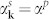 and 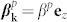 which lead to terms consistent with the mirror symmetry requirements. We further assume that the coupling between the chain and the superconductor is small compared with the chain's bandwidth so that the induced gap is much less than the other energy scales of the system. We then obtain an effective Hamiltonian by adding the self energy [equation (3)] evaluated at *ω* = 0 to the bare chain Hamiltonian. Neglecting corrections beyond nearest-neighbor pairing, we obtain the effective Hamiltonian of the chain
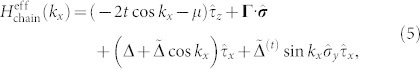
where 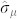 and 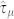 are the Pauli matrices in spin and Nambu space, respectively. The first line of the Hamiltonian describes the bare FM chain with direct inter-atom hopping *t*, chemical potential *μ*, and a Zeeman splitting 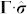 due to ferromagnetism which is comparable to the Fermi energy in the wire. The last line gives the induced superconducting gaps with both singlet (Δ and 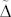) and triplet (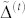) pairing potentials. The latter corresponds to a state where the triplet pairs have vanishing spin component along the *y*-axis which can gap the spin-split bands as long as **Γ** has a component in the *x*-*z* plane.

The key experimentally-relevant quantity is the local density of states (LDOS), which can be directly measured using STM. The LDOS at position *x* is defined as
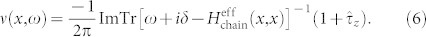
Throughout this paper we fix *t* = 10Δ and 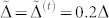, and study how the topology of the system varies as a function of *μ* and **Γ**. We emphasize that our results are generic and qualitatively independent of the precise choice of these parameters.

### Topological properties of the chain

For obtaining the topological classification of 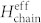 we note that it satisfies the particle-hole symmetry 
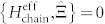
, where 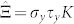 and *K* is the complex-conjugate operator. If we further assume that the *y* component of **Γ** is zero, 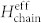 is real and hence it has the chiral symmetry 
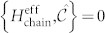
 where 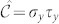. In this case, 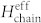 belongs to the BDI topological class and is endowed with a topological index *Q* equal to the number of zero-energy MF modes (*n* = *Q*) localized at its ends. To compute *Q*, we first rotate 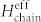 to the basis in which 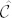 is diagonal, by 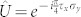, such that
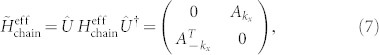
whence *Q* is computed by
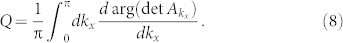


In Fig. 2 we plot *Q* against 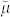 and Γ*_z_*, where **Γ** is taken as **Γ** = Γ*_z_***e***_z_* such that the chiral symmetry is respected (the chiral symmetry is respected as long as **Γ** is in the (*x*-*z*) plane), and 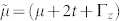 is defined from the bottom of the spin-split sub-bands. Note that for approximately 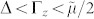, we have *Q* = 2 while for Δ, 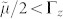 we have *Q* = 1, indicating, in both cases, the existence of MFs at the chain ends. This can be understood in the following way: in the large Zeeman spin-splitting (“half-metal”) limit, the effects of the singlet pairing terms Δ and 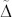 on the Bogoliubov-de Gennes spectrum are suppressed due to a large Fermi momenta mismatch between the two spin species. Then, with the triplet pairing 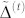, the system becomes effectively an equal-spin-pairing triplet superconductor with non-zero Δ_↑↑_ and Δ_↓↓_, which can be viewed as two copies of the Kitaev *p*-wave chain spatially superimposed on each other^30^. If 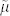 is such that both spin channels are occupied, we get *Q* = 2 (two MFs at each end of the chain), while if 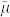 is such that only one channel is occupied, we get *Q* = 1 (an MF at each end). Since most itinerant ferromagnets are not half metals, and the induced singlet gap Δ is likely much smaller than 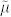, we expect that the *Q* = 2 phase is of greatest practical relevance.

In Fig. 3(a,b) we plot the LDOS at the ends of the semi-infinite FM chain as a function of 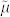 and Γ*_z_*, respectively. It can be seen that the LDOS generically has a pronounced zero-energy peak that can be accessed in STM measurements near the chain ends. For the Zeeman splitting 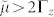 the ZBP is due to a pair of MFs localized at the same ends and protected from splitting by the topological chiral symmetry. For 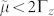 the zero-energy peak implies a single MF that should follow non-Abelian braiding statistics. No such zero-energy peak is observed in LDOS calculated for the middle of the chain, although the superconducting gap in the chain closes at the topological transitions at which the integer *Q* (and thus the number of MFs at the chain ends) changes, see the inset of Fig. 3(a,b).

## Discussion

**Figure 4:**
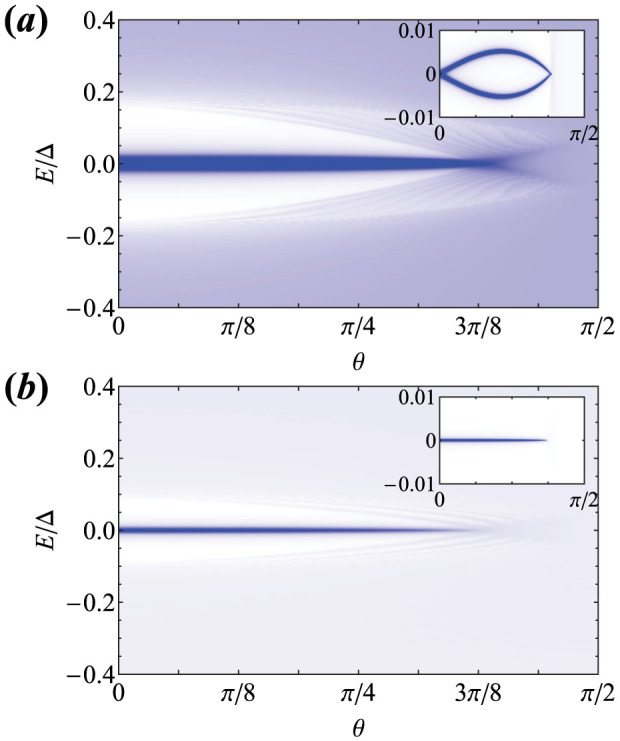
(a) The local density of states (LDOS) at one end of the semi-infinite ferromagnetic (FM) chain as a function of *θ* in the phase *Q* = 2, where 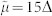 and **Γ** = 3Δ (sin *θ***e***_y_* + cos *θ***e***_z_*). Since the *y*-component of **Γ** breaks chiral symmetry, the pair of MFs at each end mix and split for finite *θ*, but the splitting is small and visible only on a small energy scale shown in the inset. (b) The LDOS at the chain end plotted against *θ* for *Q* = 1 where 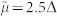 and **Γ** is the same as above. Since there is now a single Majorana fermion (MF) at each end the zero-bias peak does not split. Although the system is no longer in class BDI, it is still a class-D topological superconductor with zero or one MF at each end. We use arbitrary units for the LDOS in these plots.

While the above results demonstrate that it is not necessary to fine-tune the chemical potential or Zeeman spin-splitting to generate a zero-energy peak in LDOS (and consequently a ZBP in STM measurements) at the ends of the FM chain, a component of **Γ** perpendicular to the *x*-*z* plane breaks the chiral symmetry. To assess the effects of misalignment of the Zeeman splitting (which can, for example, be generated by a suitably applied external magnetic field), we plot in Fig. 4 the LDOS against *θ* where now we choose **Γ** = 3Δ (sin *θ***e***_y_* + cos *θ***e***_z_*). The zero-energy LDOS peak at the end of the chain splits into two peaks at finite energy by a non-zero *θ* only in the phase *Q* = 2. As *θ* is tuned up, the magnitude of the splitting first increases, then decreases, and finally vanishes with a concomitant disappearance of the localized peak. This can be understood from the observation that the *y*-component of **Γ** has an additional effect of suppressing the spectral gap of the system and since the splitting is bounded by the size of the spectral gap, the size of the splitting can never reach a large value. Therefore the splitting of the zero-energy LDOS peak due to a misalignment of the Zeeman term is always small. No such splitting should be observable in the phase with *Q* = 1. This is because in these regions of the phase diagram the ends of the chain host a single MF at each end, and thus the ZBP persists. Although the system is no longer in class BDI, it reduces to a class-D topological superconductor with zero or one MF at each end.

Before concluding, we comment on the connection to previous works. Our platform bears a superficial resemblance to proposals where the impurity band formed by a chain of magnetic impurities deposited on the surface of an *s*-wave superconductor naturally resides in a topological phase^11^,^12^, due to the self-tuned formation of a spin helix resulting from the RKKY interaction mediated by the quasiparticles in the superconductor^29^,^31^,^32^,^33^,^34^. Our system nevertheless differs from this class of proposals in several fundamental ways: (i) due to large direct hopping between the atoms in the chain, we are focused on the electronic states on the chain itself, and not on the impurity band in the superconductor; (ii) we assume a large direct exchange interaction between the impurities such that they are in a ferromagnetic arrangement and RKKY interactions play no role; and (iii) the topological state in our system follows from spin-orbit coupling in the superconductor which induces a triplet pairing term in the chain. Our work has much closer connection to previous proposals^9^,^10^ in which half-metals are proximity-coupled to spin-orbit coupled superconductor surface. These can be considered as the special case of our device where only one band of the ferromagnetic chain is occupied and a single MF is present at each end of the chain. Our crucial improvement over these schemes is that we have demonstrated that a different topological phase with two MFs at each end can be realized when both bands of the chain are occupied, which is certainly much less restrictive than requiring a half-metal chain. We also note the relation to semiconductor-superconductor heterostructure proposals^7^,^8^ where the spin-orbit coupling is in the Majorana wire itself and the spin-splitting is produced by an explicit external magnetic field in sharp contrast to our system. There has actually been one published experiment^35^ (and a related theoretical analysis^36^) involving transport studies on a somewhat related system with a Co nanowire sandwiched between superconducting electrodes although the specific MF issues of interest in the current paper were not investigated in these works. A possible experimental verification of our predictions has recently been reported in measurements of robust ZBPs at the end of ferromagnetic Fe chain on superconducting Pb^37^. While very suggestive, the actual relation to our proposal is unclear [see Ref. 38] and requires detailed modelling which is well beyond the scope of the current work.

In conclusion, we consider a FM chain deposited on the surface of a bulk *s*-wave superconductor with strong spin-orbit coupling. We establish the generic existence of a zero-energy peak in the LDOS at the ends of the chain in this system. The zero-energy peak in the LDOS should be accessible in STM experiments which should reveal a pronounced ZBP from the chain ends but not from the regions away from the ends. We show that the ZBP is due to the existence of one (odd) or two (even) MFs localized at the same end protected by a topological chiral symmetry. In this picture an STM experiment on the ends of a FM chain deposited on the surface a bulk superconductor (with strong spin-orbit coupling) will almost always show a pronounced ZBP, indicating the existence of one or two MFs at each end depending on the relative magnitudes of the ferromagnetic moment and the chemical potential.

## Methods

Here we present a derivation of the effective chain Hamiltonian. Starting from the general expression for the pseudospin states, we derive the Green's function in the orbital-spin basis. We then use this to evaluate the lowest-order self-energy correction to the chain states due to the proximity effect. Evaluated in the static limit, we add the self-energy to the bare chain Hamiltonian to obtain the effective model studied in the main text.

### Pseudospin basis

The pseudospin state is expressed in terms of the orbital-spin states as
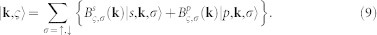
The pseudospin index *ς* = ± transforms as a spin under time-reversal (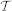) and inversion (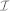) symmetries. From
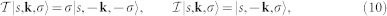

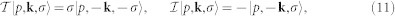
we deduce the relations obeyed by the coefficients
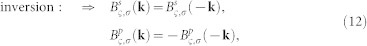

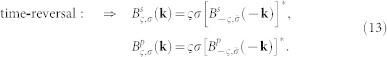
Expressed as a matrix, we have the general forms of 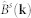 and 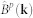

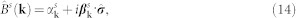

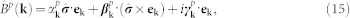
where **e_k_** is the unit vector in the direction of **k**, and the coefficients 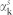, 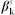, 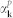, 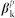, and 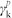 are real and even functions of **k**.

We can further constrain the forms of 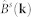 and 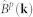 by considering mirror symmetries. We assume that the crystal has mirror planes perpendicular to the *x*, *y*, and *z* axes. We assume that the pseudospin transforms like a spin under mirror reflection, i.e.
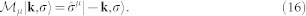
We assume that this also hold for the orbital states, except that the odd-parity orbital is odd under mirror reflection in the plane perpendicular to the *z* axis. It can then be shown that
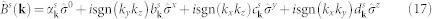

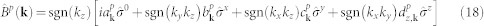
where the coefficients 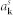, *etc.* are real functions and even under mirror reflection.

### Green's function in orbital-spin basis

Expressed in the basis 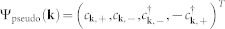, where *c***_k_**_,*ς*_ is the annihilation operator for the state with momentum **k** and pseudospin *ς*, the pseudospin Green's function of the bulk superconductor is the 4 × 4 matrix


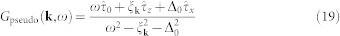
where *ξ***_k_** is the normal state dispersion, Δ_0_ is the superconducting gap, and 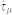 are the Pauli matrices in Nambu space. From equation (9) we have the relation
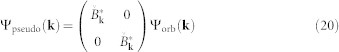
where
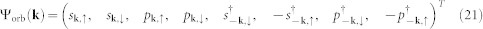
is the spinor of creation and annihilation operators in the orbital-spin basis, where *s***_k_**_,*σ*_ (*p***_k_**_,*σ*_) destroys an electron with momentum **k** and spin *σ* in the *s* (*p*) orbital, and
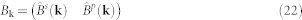
is a 2 × 4 matrix, with 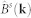 and 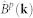 as defined above. Using equation (20), we express the Green's function in the orbital basis as
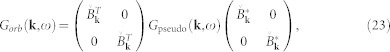
where *G*_orb_(**k**, *ω*) is an 8 × 8 matrix. It is important to note that since this Green's function is obtained from the pseudospin Green's function *G*_pseudo_(**k**, *ω*), it is only valid close to the Fermi energy. The full orbital-spin Green's function contains terms from the additional band composed from the *s* and *p* orbitals, but since this band is assumed to lie far away from the Fermi surface we ignore them.

### Proximity effect

The proximity effect in the chain due to the tunneling into the superconductor is accounted for by the self-energy
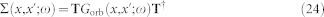
where the 4 × 8 matrix **T** describes the tunneling between the orbital-spin states of the superconductor and the ferromagnetic chain
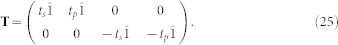
For simplicity, we approximate the Green's function of the superconductor at the surface by the bulk Green's function equation (23). This is a reasonable approximation for the conventional superconductors considered here. We hence obtain
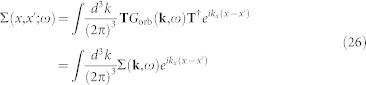
After straightforward manipulation, we find
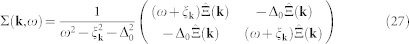
where
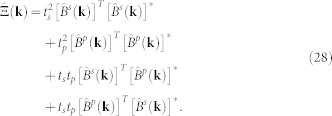
The first two terms in 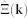 describe tunneling processes involving only one of the orbitals in the superconductor. Using the properties Eqs.(12) and (13), the matrix products here can be shown to be proportional to the unit matrix, and to be even functions of **k**. The last two terms, on the other hand, arise from tunneling processes involving both orbitals, where the matrix products are proportional to the Pauli matrices and are odd in **k**. In particular, this introduces spin-triplet pairing correlations into the ferromagnetic chain.

### Effective Hamiltonian

To obtain an effective Hamiltonian for the ferromagnetic chain including the proximity effect, we add the self-energy term evaluated at *ω* = 0 to the bare chain Hamiltonian, i.e.
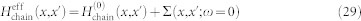
where the original chain Hamiltonian is written
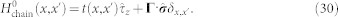
Here *t*(*x*, *x*′) = −*μδ_x_*_,*x*′_ − *t*(*δ_x_*_,*x*′+1_ + *δ_x_*_,*x*′−1_) describes the normal state dispersion, while 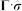 is the Zeeman splitting due to the ferromagnetism. The proximity effect renormalizes the dispersion, and also introduces an antisymmetric spin-orbit coupling, and spin-singlet and spin-triplet pairing potentials,
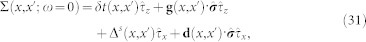
where we have the general expressions
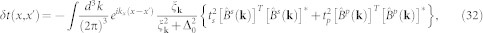

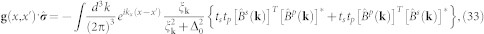

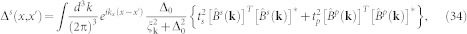

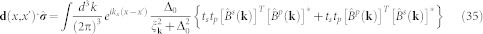
Although these expressions are quite omplicated, we can nevertheless make some generic observations. Firstly, we note that the renormalization of the dispersion and the singlet pairing potential arise only from the intra-orbital tunneling processes. On the other hand, the inter-orbital processes are responsible for the spin-orbit coupling and the triplet gap. The opposite parity of the *s* and *p* orbitals is crucial in obtaining these terms; tunneling into orbitals of the same parity could only give even-parity contributions to the self-energy. Furthermore, we observe that the induced spin-orbit coupling vector is always parallel to the triplet **d** vector, i.e. **g**(*x*, *x*′)||**d**(*x*, *x*′). We expect that the pairing terms are generally much larger than the normal-state corrections, however, due to the factor of *ξ***_k_** in the integrals of the latter. We henceforth ignore *δt*(*x*, *x*′) and **g**(*x*, *x*′) in constructing the effective Hamiltonian.

To derive a tractable model for the chain, we first assume that the proximity-effect corrections Σ(*x*, *x*′; *ω* = 0) are negligible for *x* and *x*′ further apart than nearest neighbors. We then choose coefficient matrices 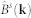 and 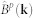. For simplicity we take 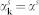, 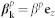, and all other coefficients vanishing in Eqs. (14) and (15); this is equivalent to 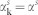, 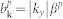, 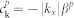 in Eqs. (17) and (18). Other choices of coefficients can only change the orientation of **d**(*x*, *x*′). We then find
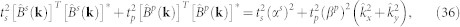

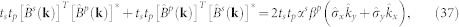
where 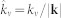. We hence obtain the gap functions
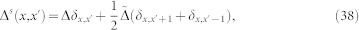

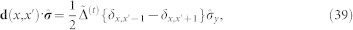
where
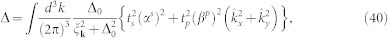

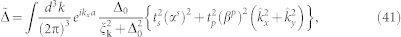

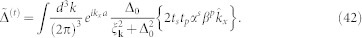
Here *a* is the lattice spacing of the chain.

Finally, we insert the pairing potentials equations (38) and (39) into equation (29) and transform to momentum space to obtain the effective Hamiltonian which is studied in the main text
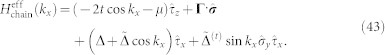


## Author Contributions

S.D.S., J.D.S. and S.T. contributed to the conceptual developments. S.T., P.M.R.B. and H.Y.H. wrote the main manuscript text and prepared the figures. All authors reviewed and edited the manuscript.
